# Three-Dimensional Quantitative Evaluation of the Segmental Functional Reserve in the Cirrhotic Liver Using Multi-Modality Imaging

**DOI:** 10.1097/MD.0000000000002719

**Published:** 2016-03-07

**Authors:** Canhong Xiang, Yingmao Chen, Mingzhe Shao, Can Li, Xin Huang, Lei Gong, Ang Li, Weidong Duan, Aiqun Zhang, Jiahong Dong

**Affiliations:** From the Center for Hepatopanreatobiliary Diseases (CX, XH, LG, AL, JD), Beijing Tsinghua Changguang Hospital, Tsinghua University Medical Center, Changping District, Beijing; Hospital & Institute of Hepato-Biliary Surgery (CX, WD, JD, AZ), Chinese PLA General Hospital, Haidian District, Beijing; and Department of Nuclear Medicine (YC, MS, CL), Chinese PLA General Hospital, Haidian District, Beijing, China.

## Abstract

To quantitatively evaluate the regional functional reserve in the cirrhotic liver and to seek related index that reflects diminished segmental liver function.

A 3D system for quantitative evaluation of the liver was used to fuse technetium-99m galactosyl human serum albumin single-photon emission computed tomography and computed tomography images from 20 patients with cirrhotic liver and hepatocellular carcinoma. A set of parameters reflecting liver function including morphological liver volume, functional liver volume, functional liver density (FLD), and the drug absorption rate constant for hepatic cells (GSA-K) was calculated. Differences in FLD and GSA-K in intrahepatic segments were compared in patients with a tumor embolus (Group Y) and those without such an embolus (Group N) in the right portal vein. Differences in FLD and GSA-K in tumor-bearing (T+ group) and tumor-free (T− group) segments in patients with no tumor embolus (Group N) were also compared. Eleven living donor liver transplantation donor served as the control group.

The FLD of the liver as a whole was significantly lower in patients with cirrhosis than in the control group (0.53 ± 0.13 vs 0.68 ± 0.10, *P =* 0.010). The FLD in segments of the right hemiliver was significantly lower than that in segments of the left hemiliver in Group Y (0.31 ± 0.21 vs 0.58 ± 0.12, *P* *=* 0.002) but not in Group N (0.60 ± 0.19 vs 0.55 ± 0.13, *P* *=* 0.294). FLD was 0.45 ± 0.17 in the T+ group and 0.60 ± 0.08 in the T− group (*P* *=* 0.008). Differences in GSA-K in intrahepatic segments were not significant. In the control group, differences in FLD and GSA-K in intrahepatic segments were not significant.

The segmental liver functional reserve can be quantitatively calculated. FLD, but not GSA-K, is an index that reflects diminished regional liver function caused by portal flow obstruction or tumor compression.

## INTRODUCTION

The functional reserve of future liver remnants is known to be closely related to morbidity and mortality after hepatectomy and it should be carefully evaluated before hepatectomy.^1–5^ In current clinical practice, regional functional reserve is mainly evaluated by gauging the parenchymal function of the liver as a whole and measuring the morphological liver volume (MLV) of individual regions.^6–10^ This comprehensive evaluation is predicated on the function homogeneity of the liver. In cases of tumor compression and/or vascular obstruction, however, liver function differs in certain portions. Whether or not the regional MLV accurately indicates liver function is unclear. Therefore, a method of efficiently evaluating regional liver function needs to be established.

Increased attention has been paid to a new method of evaluation based on 99mTc-GSA (technetium-99m galactosyl human serum albumin) scintigraphy.^11,12^ GSA is a liver-specific imaging agent and is only taken up by hepatic cells. According to the “intact hepatocyte theory,”^13^ the FLV (functional liver volume) of a region indicates the quantity of hepatic cells in that area.^14^ Indices for the liver function of each hemiliver can be calculated by combining single-photon emission computed tomography (SPECT) imaging with a CT scan.^15,16^ When an extended hepatectomy is performed, functional indices for specific anatomical areas such as individual segment of the liver are needed. To the extent known, indices for segments have yet to be described.

The current study used a 3D system for quantitative multimodality imaging of the liver (*IQQA-3D liver)* to successfully fuse CT and SPECT images and to identify the borders of intrahepatic segments. A set of parameters reflecting liver function was then calculated for the entire liver, the left hemiliver and right hemiliver, and intrahepatic liver segments. Statistical analysis was performed to compare these parameters in terms of their ability to evaluate regional liver function particularly in instances of portal flow obstruction and tumor compression.

## MATERIAL AND METHODS

One group of subjects consisted of 20 patients with liver cirrhosis and hepatocellular carcinoma (HCC). Patients consisted of 15 males and 5 females, with an average age of 53.6 ± 7.59 years (range 37–69 years). All patients underwent routine blood tests, a liver function test, and dynamic enhanced CT. Sixteen patients tested positive for hepatitis B surface antigen (HBsAg+), and none tested positive for antibodies to the hepatitis C virus (HCVAb+). Two patients had undergone transcatheter arterial chemoembolization prior to this study.

The 20 subjects had 25 HCCs in total, with an average tumor size of 6.5 cm (0.6–17.4 cm). Tumor distribution was as follows: S4 (n = 3), S5 (n = 3), S6 (n = 8), S7 (n = 5), and S8 (n = 6). Six patients had a tumor thrombus in the main trunk of the right portal vein.

Eleven living donor liver transplantation donors served as the control group. The ratio of male: female was 9:2, and the average age of donors was 30.8 ± 6.5 years (25–47 years). All donors underwent the same tests and examinations as patients with liver cirrhosis and HCC.

This study was approved by the Ethics Committee of this hospital and informed consent forms were signed by all subjects prior to participation in this study.

### ICG Test

In this study, indigo cyanogreen test (ICG) testing was done using pulse dye densitometry. Fifteen minutes after 0.5 mg/kg of the agent was administered intravenously, the plasma retention rate at 15 minutes (ICG-R15) and the plasma disappearance rate (ICG-K) were automatically calculated.

### 99mTc-GSA SPECT Images of the Liver

Images were acquired using a Siemens Symbia T6 SPECT/CT system. Before imaging, 3 mg of GSA (185MBq) (Beijing Shihong Drug Research Center, Beijing, P.R.C.) was labeled with 99mTc and allowed to stand for at least 10 minutes before injection. Patients were imaged in the supine position on the examination table with their hands placed on the sides of their head. The SPECT detectors were positioned toward the patient's liver and SPECT acquisition was started as soon as the intravenous bolus injection of the radiopharmaceutical was complete. The SPECT dual heads continuously rotated through 180 degrees with a radius 26 cm at a speed of 1 min/rotation. Twenty-six frames of tomographic images were acquired. The acquisition time per frame was 1 minute for the first 25 frames and 5 minutes for the last frame.

### The 3D Fusion of SPECT Images and CT Images

**FIGURE 1:**
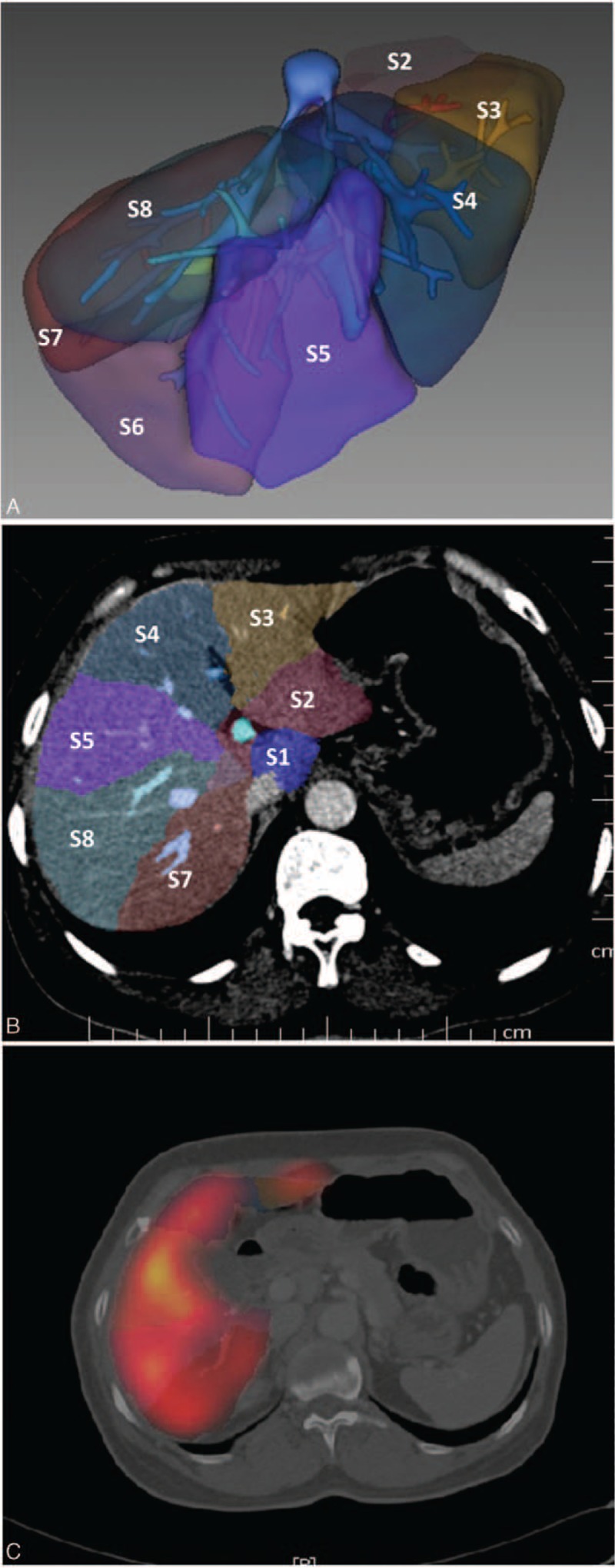
Borders of intrahepatic segments. A, Segments division according to the portal territory (3D image, S1-S8). B, Segments division according to the portal territory (transverse section image, S1-S8). C, the corresponding CT-SPECT fusion image. CT-SPECT = computed tomography–single-photon emission computed tomography.

In this study, a 3D imaging system for quantitative evaluation of the liver (*IQQA-3D liver*, Inc, Princeton, NJ) was used to fuse SPECT images with contrast-enhanced CT images of the same patient. The system uses information fusion, in which both information on joint intensity distribution and landmark correspondence were used to achieve image registration. After image fusion, branches of the portal veins and hepatic veins were identified in 3D contrast-enhanced CT images. Borders of intrahepatic segments can be automatically drawn (Figure 1A–C) and then the corresponding functional index (FLV) and MLV can be calculated. In instances of a tumor embolus in the right portal branch, the division of intrahepatic segments on the right was the result of the presence of a reconstructed middle hepatic vein, right hepatic vein, and right portal vein.^17^

### Calculation of the GSA-K

The curve for the relationship between the count rate (C(t)) and time (t) in each region of interest was fitted to C(t) = C_max_ (1 − e^−kt^) using the nonlinear least squares method. The parameter GSA-K, the drug absorption rate constant for hepatic cells, was determined.^18^

### Calculation of the MLV

Segmentation of each region was verified by the system operator, and then MLVs of all liver regions (including the entire liver, tumor-bearing and tumor-free liver parenchyma, the left and right liver lobes, and liver segments) were automatically calculated by the system.

### Calculation of the FLV

Calculation of FLV depends on the degree of Tc-GSA radioactivity in each voxel.^18^ Briefly, the maximum voxel count in the 26th frame was determined in images of the entire liver. Three-dimensional contours of each volume of interest (VOI) were drawn at a threshold of 54% and 80% of the maximum voxel count. The total count and average count in each VOI and the volume of each VOI were determined. The VOIs were then copied to the first 25 frames of the images. The count rate-time curve was plotted for VOIs in 26 frames. The thickness of each voxel with a count above 80% of the maximum voxel count was estimated to be 1.08 cm, and the thickness of each voxel with a count that was from 54% to 80% of the maximum voxel count was estimated based on the overall voxel count. Voxels with a count below 54% of the maximum voxel count were considered to be background.

### Calculation of the Functional Liver Density

FLV differs in individuals and in regions, thus precluding a simple comparison. Accordingly, a new index, functional liver density (FLD), was created. The FLD of the hepatic parenchyma is calculated as the ratio of the FLV to the MLV for the region in question. FLD therefore indicates the extent of GSA uptake and can be used to evaluate and compare the regional functional reserve.

### Statistical Analysis

All data are expressed as mean ± SD. Correlations between FLV, GSA-K, FLD, and the results of liver function tests including total bilirubin, ICG-K, albumin, International Normalized Ratio (INR), and the Child-Pugh score were analyzed using a standard Pearson correlation analysis. Indices for each group were compared using the student *t* test. Parameters measured in intrahepatic segments were compared using repeated measures analysis of variance. A *P* < 0.05 was considered to represent a significant difference. The software SPSS 15.0 (SPSS Inc, Chicago, IL) was used for statistical analysis.

## RESULTS

### Functional Reserve Indices for the Liver As a Whole

In patients with cirrhosis, the hepatic parenchyma had an MLV of 1218.9 ± 306.8 mL (796.3–2617.2 mL), an FLV of 649.0 ± 169.9 mL (412.6–983.5 mL), an FLD of 0.53 ± 0.13 (0.28–0.76), and a GSA-K of 0.15 ± 0.03 (0.10–0.21) (Table 1).

**TABLE 1:**
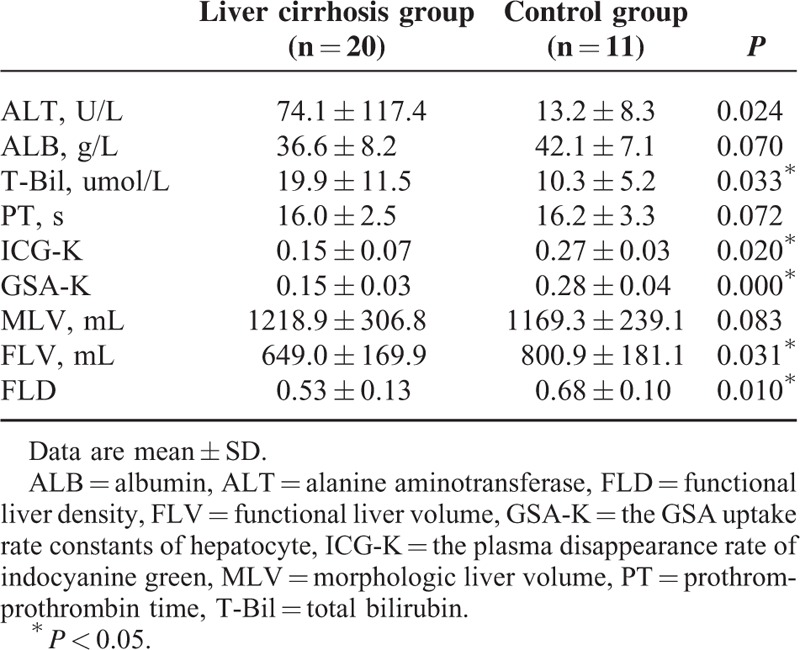
Comparison of the parameters between patients in liver cirrhosis group and control group

GSA-K closely correlated with the ICG-K (*P* < 0.0001), child-pugh score (*P* = 0.006), and INR (*P* < 0.0001). FLV and FLD were not correlated with liver function indices (Table 2).

**TABLE 2:**
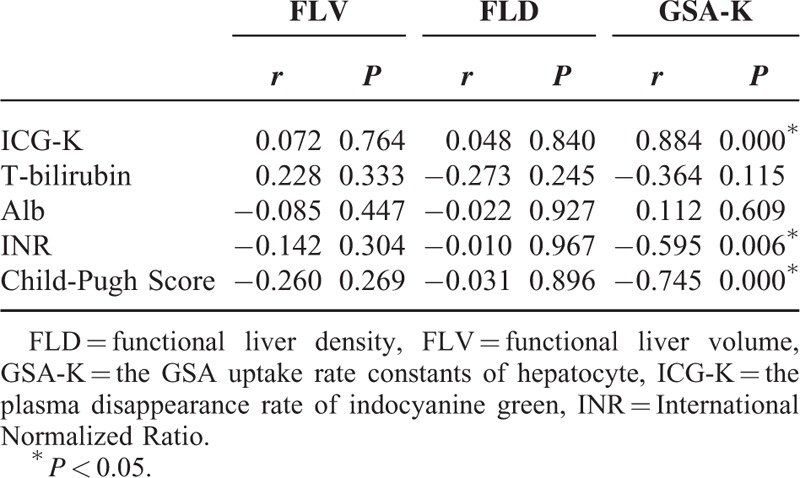
Correlation between the indices of FLV, FLD, 99mTc-GSA (GSA-K) and the liver function index in liver cirrhosis group

Tumor had an MLV of 330.2 ± 389.7 mL (4.0–1588.2 mL), an FLV of 19.7 mL (0.0–80.6 mL), and an FLD of 0.04 ± 0.05 mL (0–0.14 mL).

In the control group, the hepatic parenchyma had an MLV of 1169.3 ± 239.1 mL (869.0–1639.2 mL), an FLV of 800.9 ± 181.1 mL (560.3–1218.3 mL), an FLD of 0.68 ± 0.10 (0.51–0.80), and a GSA-K of 0.28 ± 0.04 (0.22–0.35) (Table 1).

**FIGURE 2:**
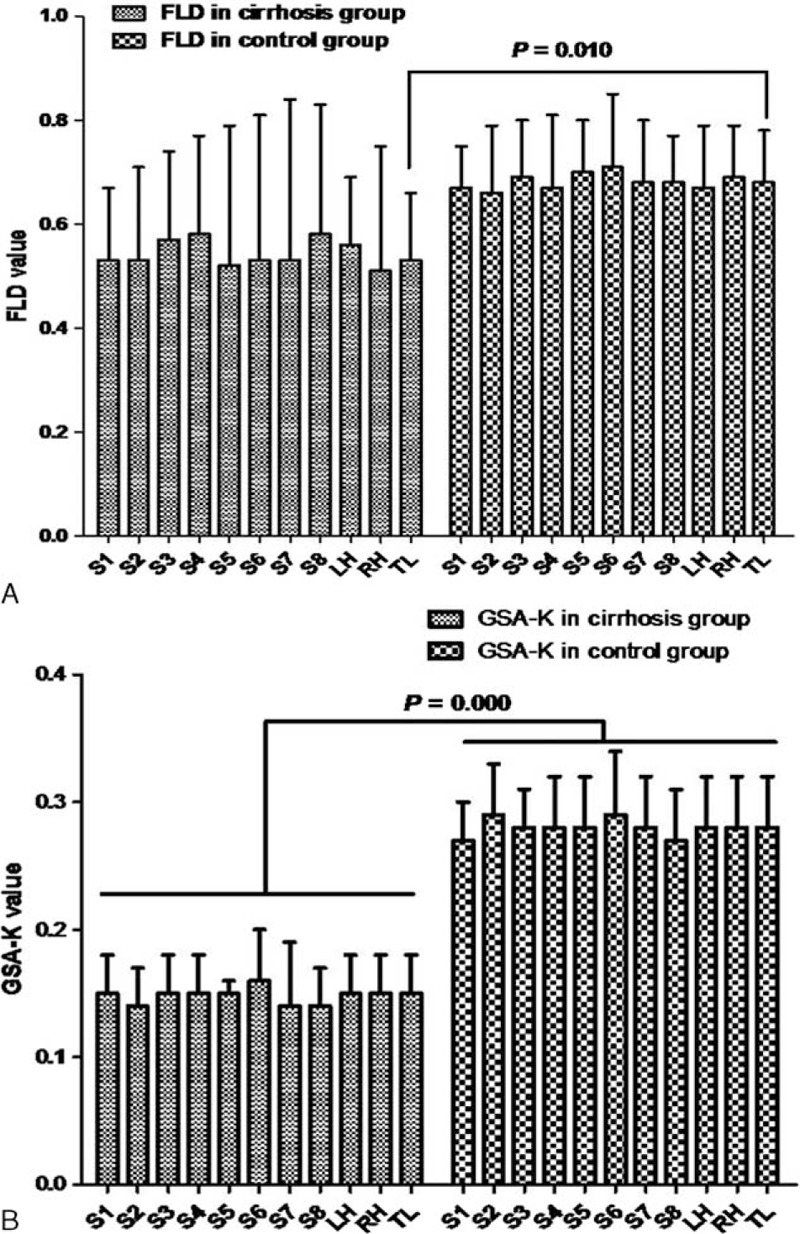
Functional indices in cirrhosis group and control group. A, FLD value in cirrhosis group and control group. B, GSA-K value in cirrhosis group and control group. (Data are mean ± SD.) FLD = functional liver density, GSA-K = the GSA uptake rate constants of hepatocyte, LH = left hemiliver, RH = right hemiliver, TL = total liver.

There were significant differences in the FLD (*P* = 0.010) and GSA-K (*P* < 0.0001) of patients with cirrhosis and the control group (Figure 2A and B).

### Evaluation of the Segmental Liver Function Reserve

Functional indices for each hemiliver and segments in patients with cirrhosis are shown in Table 3a and Figure 2A and B. Differences in FLD and GSA-K in intrahepatic segments were not significant (*P* > 0.05). Functional indices for both hemilivers and liver segments in the control group are shown in Table 4 and Figure 2A and B. Differences in FLD and GSA-K in intrahepatic segments were not significant (*P* > 0.05).

**TABLE 3:**
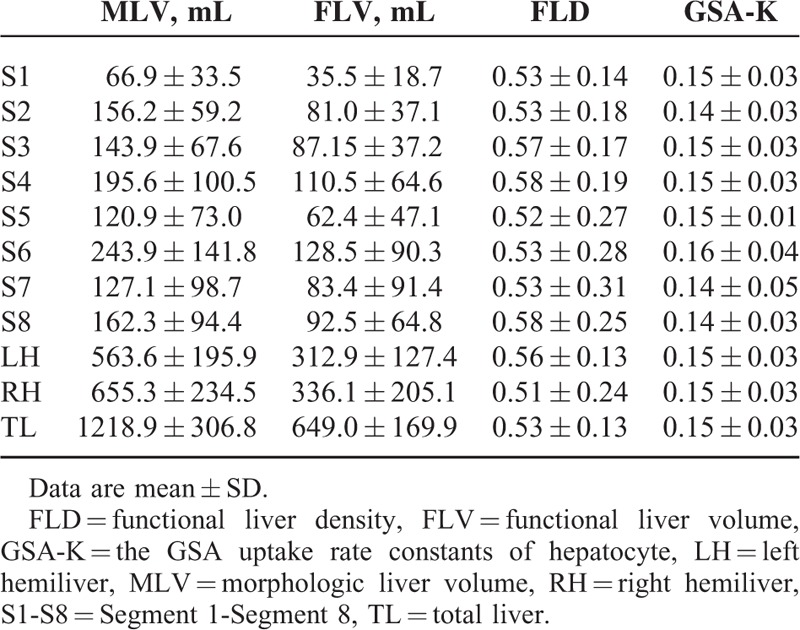
Results of functional parameters of intrahepatic anatomic areas in liver cirrhosis group

**TABLE 4:**
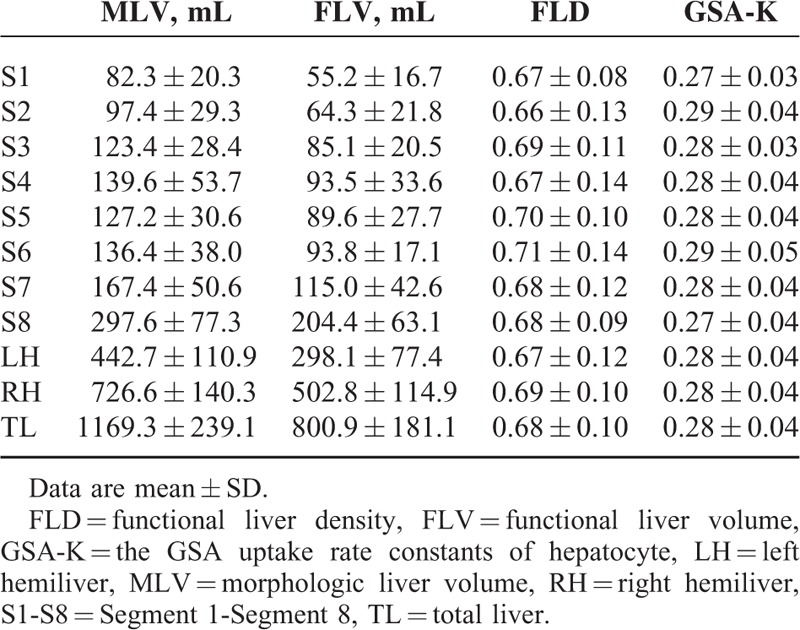
Results of functional parameters of intrahepatic anatomic areas in control group

### The Effects of Portal Flow on Segmental Liver Function in Patients With Cirrhosis

**FIGURE 3:**
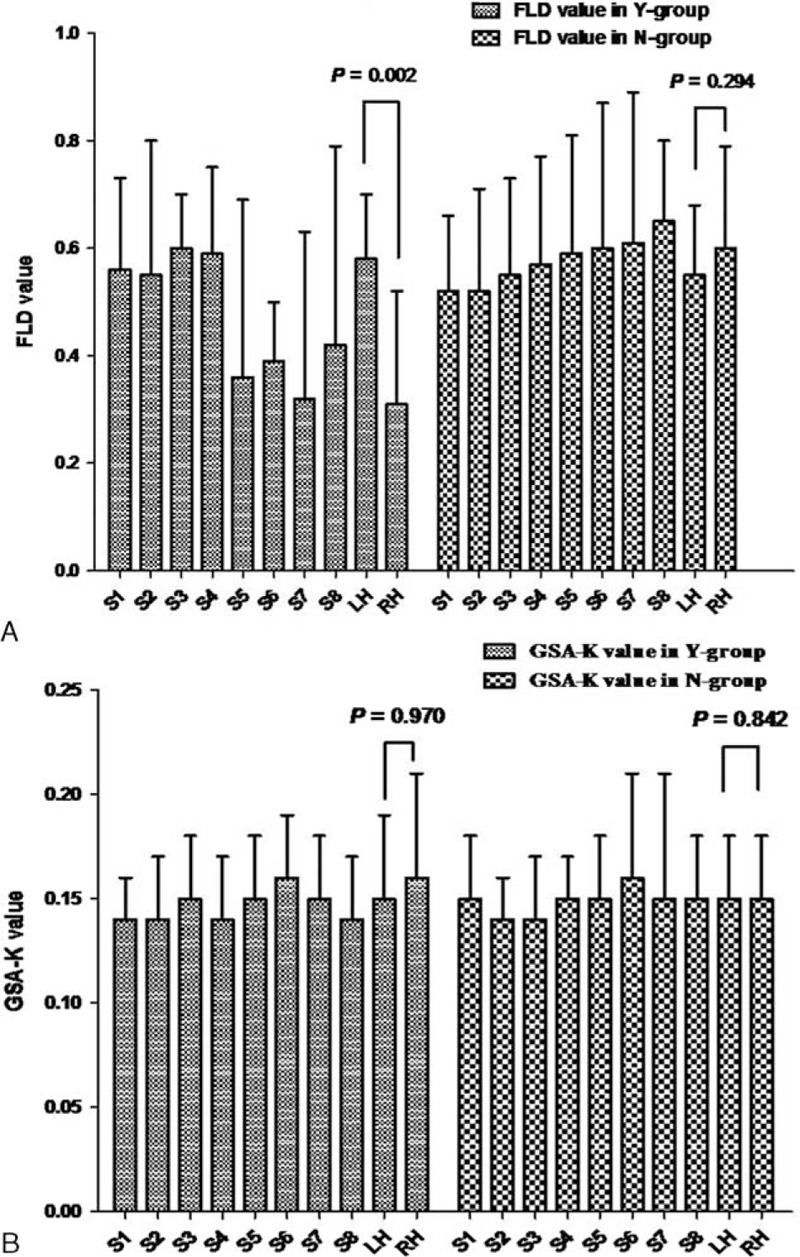
Functional indices in Y group and N group. A, FLD value in Y group and N group; B, GSA-K value in Y group and N group. FLD = functional liver density, GSA-K = the GSA uptake rate constants of hepatocyte, LH = left hemiliver, N group = patients without tumor thrombus in the main trunk of right portal vein, RH = right hemiliver, TL = total liver, Y group = patients with tumor thrombus in the main trunk of right portal vein.

To analyze the effects of portal flow obstruction on regional liver function, patients with cirrhosis were divided into 2 groups depending on whether a tumor thrombus was present in the right portal vein (Group Y, n = 6) or not (Group N, n = 14). The FLD and GSA-K for both the groups are shown in Figure 3A and B. The FLD and GSA-K for the right hemiliver and left hemiliver were compared. In Group Y, the mean FLD of the right hemiliver was 0.31 ± 0.21 and the mean FLD of the left hemiliver was 0.58 ± 0.12 (*P* = 0.002). In Group N, the mean FLD of the right hemiliver was 0.60 ± 0.19 and the mean FLD of the left hemiliver was 0.55 ± 0.13 (*P* = 0.294). In Group Y, differences in the FLD of segments of the right hemiliver and left hemiliver were not significant (*P* = 0.503), but the FLD for segments of the right hemiliver (S5-S8) was far smaller than the FLD for segments of the left hemiliver (S1-S4) (*P* = 0.029) (Figure 3A). In Group N, differences in the FLD for segments of the right hemiliver and left hemiliver were not significant (Figure 3A).

In Group Y, the mean GSA-K in the right hemiliver was 0.16 ± 0.05 and that in the left hemiliver was 0.15 ± 0.04 (*P* = 0.970). In Group N, the mean GSA-K in the right hemiliver was 0.15 ± 0.03 and that in the left hemiliver was 0.15 ± 0.03 (*P* = 0.842). Differences in the FLD in segments of the right hemiliver and left hemiliver were not significant (Figure 3B).

### The Effects of Tumor Compression on Segmental Liver Function

In order to ascertain the effects of tumor compression on regional liver function and to avoid including the effects of portal flow, patients in Group N were analyzed further. The intrahepatic segments in each same patient were divided into 2 groups: tumor-bearing segments (T+ group) and tumor-free segments (T− group). Differences in FLD and GSA-K in these 2 groups were then compared. FLD was 0.45 ± 0.17 in the T+ group and 0.60 ± 0.08 and in the T− group; the difference in FLD was significant (*P* *=* 0.008). GSA-K was 0.15 ± 0.03 in the T+ group and 0.15 ± 0.03 in the T− group. The difference in GSA-K was not significant (*P* *=* 0.477).

## DISCUSSION

Together with SPECT imaging, 99mTc-GSA scintigraphy can be used to evaluate the functional reserve of each hemiliver.^15,18,19^ Smaller segmental areas require more precise fusion of CT and SPECT images, but techniques to do so have yet to be reported. The challenge of accurately fusing 2 image sets, particularly when the images are acquired with different devices and at different times, is how to accommodate body motion and breathing and nonrigid liver deformation caused by such motion. Numerous techniques for crossmodality image fusion have been described in the literature, such as positron emission tomography and CT fusion.^20^ One widely adopted approach for crossmodality image fusion is to use mutual information in conjunction with a deformable model to compute a deformation field using the 2 imaging modalities.^21^ One major shortcoming of this approach is that it is based on the statistical measurement of concordance between the overall intensity distribution in CT and SPECT images in which all image pixels are treated equally. Therefore, statistical measurement may be greatly biased by intensity information from anatomical structures that are not of interest. Another widely used method of crossmodality image registration involves the use of landmarks to facilitate deformable registration.^22^ In 99mTc-GSA SPECT images, however, there are few reliable landmarks available, thus affecting the accuracy and effectiveness of such an approach. Existing approaches are not suitable for fusion of 99mTc-GSA SPECT and CT images to assess regional liver function. The *IQQA-3D Liver* system uses a hybrid approach to overcome the problems with existing approaches. The system uses both landmark-based and intensity-based information to define a crossmodality registration metric. Furthermore, knowledge-based information weighting is used whereby the weight of each category of information input in the cost function is automatically assigned and adapted depending on its correlation with the area of interest. Therefore, the system can provide robust registration that is unaffected by body motion and breathing. The current study successfully achieved 3D fusion of CT images and SPECT images, and the indices of FLD and GSA-K were used to evaluate the functional reserve of intrahepatic segments.

FLD is a new index that represents functional density, that is, the quantity of functional liver cells in a given region according to the “intact hepatocyte theory.”^13^ The density of functional liver cells is known to decrease in the cirrhotic liver. The current study found that FLD was far smaller in patients with cirrhosis than in the control group. In a study to predict hepatic functional reserve before surgery, Mitsumori et al^19^ found that the predictive value of FLV based on GSA scintigraphy was superior to the MLV provided by CT volumetry, suggesting that the latter indices were hampered by tumor compression of the surrounding parenchyma and vessels. The current study confirmed this conjecture and FLD is an index that can quantitatively measure disparities in regional liver function as mentioned earlier. When patients were grouped depending on whether a tumor embolus was present, the FLD of the affected hemiliver was far smaller than the FLD for the unaffected hemiliver. The same held true for involved segments. This finding suggests that the FLD is highly responsive to changes in the portal flow in affected intrahepatic areas. The FLD for tumor-bearing segments was also smaller than the FLD for the tumor-free segments, suggesting that the FLD reflects diminished regional function due to tumor compression.

FLD can also be used as an index to ascertain disproportionality between the “volume” and “function” of an unembolized lobe after selective portal vein embolization. Uesaka et al^23^ studied the function of the left lobe after right portal vein embolization (PVE) by measuring the quantity of ICG excreted in bile drainage. They found that the increase in ICG excretion in the left liver was greater than the gain in MLV. Other researchers used GSA scintigraphy to examine the function of individual lobes after selective portal vein embolization, and they similarly found that the increase in FLV was greater than the gain in MLV.^16^ In other words, the FLD of the unembolized lobe increased after PVE. Bilateral percutaneous transhepatic biliary drainage is an invasive procedure and not practical in most clinical settings, but GSA scintigraphy is a noninvasive examination and FLD is an index that is easy to use.

GSA-K is determined based on a time-index curve. The higher the GSA-K, the quicker the uptake of GSA. In other words, a higher GSA-K reflects increased liver function. The current study found that GSA-K closely correlated with functional indices for the liver as a whole, and this finding agrees with the results of a previous study.^24^ GSA-K can be used as an index to gauge the degree of liver cirrhosis. Furthermore, GSA-K was found to differ between individuals but was equivalent in different segments of the liver. GSA-K was also unaffected by portal flow or tumor compression. To the extent known, the current study is the first to report this finding.

FLD decreased dramatically while the GSA-K remained unchanged in segments with decreased portal flow. This phenomenon is also apparent when studying PVE (unpublished data). Although the number of functional liver cells decreases in an affected segments, GSA appears to retain its binding affinity for asialoglycoprotein receptors and this affinity takes a long time to diminish.

Many hepatobiliary centers use a combination of ICG testing and MLV in their decision tree to evaluate regional functional reserve.^25–28^ This combination consists of 2 components, “function” and “volume,” implying the homogeneity of functional reserve in the liver as a whole. However, portal flow obstruction and tumor compression can diminish the segmental functional reserve, resulting in a lower regional FLD. If an affected segment is slated for resection, the predicted percentage of functional parenchyma that will be resected will be high and thus the future liver remnant will represent a small portion of the entire liver or standardized liver volume. Such results may rule out surgery for patients with marginal functional reserve.^29^ This should be taken into account during the surgical decision-making process.

## CONCLUSIONS

This study successfully developed a new method of 3D quantitative evaluation of the regional functional reserve of intrahepatic segments using multimodality image fusion. FLD, but not GSA-K, is an index that reflects diminished regional liver function caused by portal flow obstruction or tumor compression.
